# Supplementary material for: Effects and safety of vibration therapy in knee osteoarthritis rehabilitation: an umbrella review of systematic reviews
Source: PeerJ. 2025 Dec 19;13:e20455. doi: 10.7717/peerj.20455 (PMC12721121; doi:10.7717/peerj.20455)
Supplement: Supplemental Information 1 [file peerj-13-20455-s001.docx]

**Intended Audience**
This umbrella review is intended for a multidisciplinary audience that includes physiotherapists, rehabilitation specialists, clinicians managing knee osteoarthritis (KOA), researchers in musculoskeletal and rehabilitation sciences, and healthcare policymakers. The synthesized evidence aims to guide clinical decision-making, inform the development of standardized vibration therapy protocols, and identify gaps for future research to optimize nonpharmacological interventions in KOA rehabilitation.
